# Relationship between the Concentrations of Heavy Metals and Bioelements in Aging Men with Metabolic Syndrome 

**DOI:** 10.3390/ijerph120403944

**Published:** 2015-04-10

**Authors:** Iwona Rotter, Danuta Kosik-Bogacka, Barbara Dołęgowska, Krzysztof Safranow, Anna Lubkowska, Maria Laszczyńska

**Affiliations:** 1 Independent Laboratory of Medical Rehabilitation, Pomeranian Medical University, Żołnierska 54, 71-210 Szczecin, Poland; E-Mail: iwrot@wp.pl; 2 Department of Biology and Medical Parasitology, Pomeranian Medical University, Powstańców Wielkopolskich 72, 70-111 Szczecin, Poland; E-Mail: kodan@pum.edu.pl; 3 Department of Laboratory Diagnostics, Pomeranian Medical University, Powstańców Wielkopolskich 72, 70-111 Szczecin, Poland; E-Mail: barbara.dolegowska@pum.edu.pl; 4 Department of Laboratory Diagnostics, Poznan University of Medical Sciences, Szamarzewskiego 82/84, 60-569 Poznań, Poland; E-Mail: barbara.dolegowska@pum.edu.pl; 5 Department of Biochemistry and Medical Chemistry, Pomeranian Medical University, Powstańców Wielkopolskich 72, 70-111 Szczecin, Poland; E-Mail: chrissaf@mp.pl; 6 Department of Physical Medicine and Functional Diagnostics, Pomeranian Medical University, Żołnierska 54, 71-210 Szczecin, Poland; E-Mail: annalubkowska@gmail.com; 7 Department of Histology and Developmental Biology, Pomeranian Medical University, Żołnierska 48, 71-210 Szczecin, Poland; E-Mail: maria@laszczynska.pl

**Keywords:** metabolic syndrome, heavy metals, magnesium, tungsten, bioelements

## Abstract

Heavy metals may exacerbate metabolic syndrome (MS) but abnormal serum concentrations of bioelements may also co-exist with MS. The primary aim of the study was to assess the relationship of blood heavy metal and bioelement concentrations and MS, in men aged 50–75 years. Heavy metals—lead (Pb), cadmium (Cd), mercury (Hg), arsenic (As), tungsten (W), Macroelements—magnesium (Mg) and calcium (Ca), and microelements—iron (Fe), zinc (Zn) copper (Cu), chromium (Cr), molybdenum (Mo), selenium (Se) and manganese (Mn), body mass index (BMI), waist to hip ratio (WHR), abdominal circumference (AC) and blood pressure (BP), total cholesterol (TCh), high-density lipoprotein (HDL), low-density lipoprotein (LDL), triglyceride (TG), fasting plasma glucose (FPG), insulin, and Homeostasis Model Assessment—Insulin resistance (HOMA-IR). The men with MS showed statistically significant higher Zn and lower Mg concentrations. Those with diabetes had higher Ca concentration and lower Mg concentration. Cr and Mn concentrations were significantly higher in obese men. The participants with hypertension had lower Mg concentration. We found statistically significant positive correlations (W-TCh, W-LDL, Mg-TCh, Mg-LDL, Ca-TCh, Ca-LDL, Ca-insulin, Ca-HOMAR-IR, Zn-TG, Zn-insulin, Zn-HOMA-IR, Cu-BP systolic, Mn-BMI, Mn-AC, Mn-WHR, Mn-insulin, Mn-HOMA-IR, Se-TCh, Se-LDL, Se-TG, Se-insulin, Se-HOMA-IR, Cr-TCh, Cr-HDL, Cr-LDL, Cr-TG) and negative correlations (Cd-insulin, Hg-WHR, W-insulin, W-HOMA-IR, Mg-BMI, Mg-AC, Mg-WHR, Mg-BP systolic, Mo-insulin, Mn-HDL). Tungsten may contribute to lipid disorders. Magnesium appears to play the protective role in the occurrence of metabolic disorders. Microelements Mn, Cr and Se may intensify MS.

## 1. Introduction

MS is a grouping of factors that increases the risk for the development and progression of type II diabetes mellitus (TIIDM) and cardiovascular disease [1]. The criteria for identifying MS include three of five markers: abdominal obesity, impaired carbohydrate metabolism, high blood pressure and dyslipidemia (elevated levels of triglycerides and decreased levels of high-density lipoprotein (HDL)). In developed countries, MS occurs in approximately 25% of the middle-aged population [2]. In Poland, this problem affects 28.4% of men aged 40–59 years and 34.5% of men aged 60–74 [3].

Environmental exposure to heavy metals commonly used in industry, including lead (Pb), arsenic (As, a metalloid) and cadmium (Cd), increases the occurrence of diabetes and cardiovascular diseases [4,5,6,7]. Although tungsten (W) is considered to be a relatively non-toxic metal, it is known for its thrombogenic and proinflammatory action [8]. Its effects are also antagonistic to copper (Cu). Tungsten may interfere with the biological effects of molybdenum, an essential trace metal found of several enzymes [9,10]. On the other hand, some studies in animal models demonstrate that W intake contributes to weight loss and improves insulin sensitivity [11,12], although this weigh loss may be secondary to low grade toxicity. However, research in this area is scarce.

More commonly, the relationship between the prevalence of MS and the concentrations of macro- and micro-elements vital to life such as magnesium (Mg), iron (Fe), zinc (Zn), Cu, selenium (Se), manganese (Mn) and chromium (Cr). Magnesium is a cofactor of many enzymes involved in the metabolism of lipids and carbohydrates, and hypomagnesaemia often coexists with MS, TIIDM, insulin resistance and hypertension (HA) [13,14,15,16], although the role of Mg supplementation in the normalization of the metabolic profile is equivocal [13,17,18]. Many studies confirm the link between high Fe concentration and the presence of individual components of MS [19,20,21,22,23]. Research on relationships between Zn and Cu concentrations and the occurrence of TIIDM show that in type 2 diabetics, Zn concentration is significantly lower and Cu concentration is elevated compared to healthy participants [24,25]. Lower serum Cu may be associated with development of diabetic complications [26], while Zn supplementation may prevent the development of MS and diabetes [27]. It still has not been explained whether there is a clear relationship between the concentrations of these elements and MS, TIIDM and TIIDM complications [27]. 

Selenium exhibits antioxidant activity but its impact on metabolic disorders is inconclusive. Some researchers suggest that the antioxidant properties of Se may prevent metabolic disorders [28,29]. On the other hand, there exists data which indicates the negative impact of Se on some parameters of lipid and carbohydrate metabolism [30,31] so there may be a narrow window of optimum concentration. Manganese and chromium are components or activators of some enzymes, mostly antioxidants, and play an important role in the metabolism of carbohydrates and lipids, leading to the normalization of the synthesis and secretion of insulin and, therefore, having protective effects against the occurrence of MS [32,33].

The aim of this study was to assess the relationship between the concentrations of heavy metals (Pb, Cd, mercury—Hg, As, W) and bioelements (Mg, calcium—Ca, Fe, Zn, Cu, Cr, Mo, Se, Mn) and the prevalence of MS and concomitant metabolic disorders, such as diabetes and obesity, in men aged between 50–75 years. Furthermore, the aim was to assess the relationship between the concentrations of heavy metals and bioelements and the lipid parameters concentrations, including total cholesterol (TCh), high-density lipoprotein (HDL), low-density lipoprotein (LDL), triglyceride (TG), carbohydrate metabolism parameters: fasting plasma glucose (FPG), level, insulin, insulin resistance (IR), as well as anthropometric parameters: Body Mass Index (BMI), waist to hip ratio (WHR), abdominal circumference (AC) and blood pressure (BP).

Older men in Poland have a higher likelihood of working in or near to heavy industrialization at times when pollution was not well controlled, and are generally at higher risk of MS rather than women. Given the fact that oxidative stress plays a role in the pathogenesis of MS [34,35,36], we examined the hypothesis that higher concentrations of toxic metals and abnormal concentrations of bioelements may disorders contribute to MS, particularly in aging men.

## 2. Material and Methods 

The study comprised 313 men aged 50–75 years (mean age 61.3 years ± 6.3) who had volunteered after receiving information about the study from family doctors at primary health care centres operating in Szczecin (north-western Poland). Smokers were included but men were excluded if they were receiving steroids or neuroleptics, were hypothyroid or hyperthyroid, and were undergoing cancer treatment. The men were not professionally or knowingly exposed to toxic levels of heavy metal. The men were not professionally or knowingly exposed to toxic levels of heavy metals and a medical questionnaire showed that none of the participants exceeded a daily alcohol intake of 40 g. According to the WHO guidelines, exceeding this threshold in men is considered to be hazardous to health. The subjects were men from north-western Poland, which is not an industrialized area. The men did not work in industry, and most of them were pensioners, while the others worked in services.

This study conforms to the principles outlined in The Declaration of Helsinki as revised in 2008. The study ran from March 2013 to February 2014 and was approved by the Bioethics Committee of the Pomeranian Medical University in Szczecin (KB-0012/159/12). The participants who participated in the study were informed about the course of the study gave informed consent (written) to take part in it. 

The men were surveyed for demographic data and the presence of chronic diseases. The blood pressure, weight, height, waist and hip circumference were measured. Body mass index (BMI) and waist-to-hip ratio (WHR) were calculated. Body weight of the participants averaged 87 ± 15 kg, BMI 28.3 ± 7.7 kg/m^2^, waist 102 ± 12 cm and WHR 0.99 ± 0.06. The average systolic blood pressure was 135 ± 20 mmHg and diastolic blood pressure was 84 ± 20 mmHg.

Fasting venous blood samples were taken from mornings, between 7.30 a.m., to 9.00 a.m. For biochemical analyses and the determinations of bioelements (Mg, Fe, Zn, Cu, Ca, Mo, Cr, Se, Mn), blood was collected into a tube containing a coagulator and gel separator, and was then centrifuged. To determine the concentration of heavy metals (Pb, Cd, Hg, As, W), blood was collected into tubes with an anticoagulant (EDTA). We stored the sampled blood and serum at −70 °C.

In order to determine the concentration of toxic metals and bioelements, we used inductively coupled argon plasma optical emission spectrometry (ICP OES), using an Optima 2000 DV camera manufactured by Perkin Elmer (Norwalk, CT, USA), following decomposition and removal of organic matrix from the blood samples in a microwave mineralizer manufactured by Anton Paar (Vienna, Austria). After the addition of HNO_3_ and H_2_O_2_, we placed the blood or serum samples in a microwave oven which allowed a continuous monitoring of temperature and pressure in each vessel. Sample mineralization was performed according to the “Meat” procedure. To calibrate the method, we used certified multi-element standard CERTIPUR® ICP multi-element standard VIII (Merck KGaA, Darmstadt, Germany). The standard solutions were supplemented with mineralization reagents at concentrations equal to those in the mineralized samples. The emitted radiation intensity was always measured using the longer axial (along the plasma) optical path of the spectrometer. In order to verify the accuracy of the analytical method, we made the determinations of two certified reference materials (whole blood and serum): Seronorm^TM^ Trace Elements of Whole Blood L-2 and Trace Elements Seronorm^TM^ Serum L-2, produced by SERO AS (Billingstad, Norway). The levels of individual elements in the blood and serum are shown in Table 1.

We determined the concentrations of FPG, TCh, TCh, HDL, LDL and TG in the samples, employing the spectrophotometric method and reagent kits (Biolabo, Aqua-Med, Łódź, Poland). Insulin levels were determined by ELISA method using reagent kits (DRG Medtek, Warsaw, Poland). We calculated insulin resistance IR in patients without diabetes according to the formula: HOMA-IR = FPG (mmol/L) × fasting insulin levels (µU/mL) / 22.5. 

Metabolic syndrome (MS), which was diagnosed according to the 2005 criteria of the International Diabetes Federation (IDF) [37]. According to IDF guidelines for the diagnosis of MS in men in Europe, MS is indicated by an abdominal circumference ≥ 94 cm, and two of the following abnormalities: HDL cholesterol <40 mg/dL (1.03 mmol/L) or the treatment of dyslipidemia, TG ≥ 150mg/dL (1.7 mmol/L ) or the treatment of dyslipidemia, FPG ≥ 100 mg/dL (5.6 mmol/L) or the treatment of diabetes, systolic BP ≥ 130 mmHg, diastolic BP ≥ 85mmHg or antihypertensive treatment.

**Table 1:** The concentrations of elements in the whole blood and serum of participants.

Material	Element	Measurement (mcg/L) and SD
Whole blood	Pb	310.4 ± 4.3
	Cd	5.60 ± 0.34
	Hg	16.7 ± 1.4
	As	15.3 ± 1.1
	W	6.01 ± 0.1
Serum	Mg	40.8 ± 1.0
	Fe	2.18 ± 0.07
	Ca	136.3 ± 3.5
	Cu	3289.0 ± 55.0
	Zn	2351.0 ± 42.0
	Se	156.9 ± 5.1
	Mo	0.89 ± 0.06
	Mn	20.0 ± 1.4
	Cr	4.76 ± 0.38

Statistical analysis was carried out using the program Statistica PL (Statsoft, Tulsa, OK, USA). The distribution normality was examined using a Shapiro-Wilk test. Due to the non-parametric distribution of results, intergroup comparisons were performed using the Mann-Whitney U test. The correlations between the analyzed variables were calculated with Spearman’s rank correlation coefficient (r_s_). In addition, in order to assess the possible impact of cigarette smoking on the incidence of MS in the study group, a logistic regression model was used.

## 3. Results

In the group of 313 men, TCh concentrations were in the range 2.48–10.76 mmol/L) (mean 5.66 ± 1.47), HDL 0.49–2.46 mmol/L (1.27 ± 0.31), LDL 0.52–8.84 mmol/L (mean 3.67 ± 1.40), and TG 0.42–6.56 mmol/L (1.63 ± 0.95). The parameters of carbohydrate metabolism were determined in 256 participants without diabetes. The FPG concentrations ranged 2.94–13.17 mmol/L (mean 5.94 ± 1.22), the mean insulin level was 14.8 ± 7.65 μLU/mL (1.22–55.42), and HOMA-IR ranged 0.31–15.81 (mean 3.95 ± 2.22). MS was determined in 161 men (51.44%) and TIIDM in 17.57% of the men (n = 55), hypertension in 54.63% (n = 171), overweight (BMI: 25–29.9 kg/m^2^) in 164 (52.39%), obesity (BMI ≥ 30 kg/m^2^) in 27.79% of the men (n = 87). Statins were being taken by 49 (15.65%) of the men. All participants with hypertension were being treated with antihypertensive drugs.

Table 2 presents the concentrations of heavy metals (Pb, Cd, Hg, As and W) and bioelements (Mg, Ca, Fe, Zn, Cu, Cr, Mn, Mo, Se) in men with MS and without MS. Only Mg concentration was significantly lower and Zn concentration significantly higher in men with MS compared to those without MS. There were no other statistically significant differences in the concentrations of heavy metals or bioelements between the groups. 

Table 3 presents the concentrations of heavy metals and bioelements in men with TIIDM. Only Mg concentration was statistically significantly lower and Ca concentration statistically significantly higher in the diabetic patients compared to the healthy participants. 

**Table 2:** Concentrations of heavy metals and bioelements in men with and without metabolic syndrome (MS) (AM, arithmetic mean; SD, standard deviation; Med—median; *p*: probability for trend).

Metal	MS (n = 161)			Without MS (n = 152)			*p*
	AM (SD)	Med	Range	AM (SD)	Med	Range	
**Heavy Metals**							
Pb (mcg/L)	75.17 (21.4)	73.00	24.3–161.5	73.82 (21.7)	71.05	28.0–138.5	0.46
Cd (mcg/L)	1.55 (0.32)	1.59	0.59–3.06	1.53 (0.34)	1.53	0.62–2.35	0.41
As (mcg/L)	7.83 (1.59)	7.80	3.45–15.2	7.83 (1.61)	7.97	2.48–12.5	0.67
Hg (mcg/L)	4.59 (0.92)	4.61	0.15–9.10	4.51 (0.80)	4.46	2.72–7.90	0.21
W(mcg/L)	0.62 (0.01)	0.62	0.28–1.11	0.62 (0.01)	0.62	0.42–0.83	0.67
**Bioelements**							
Mg (mg/L)	20.6 (2.05)	20.6	10.0–26.3	21.28 (2.37)	21.15	13.8–37.5	0.02
Ca (mg/L)	95.06 (6.87)	95.5	53.5–117	95.47 (8.06)	95.25	75.5–159.6	0.38
Fe (mg/L)	1.19 (0.45)	1.10	0.28–3.40	1.23 (0.43)	1.47	0.29–3.0	0.35
Zn (mg/L)	0.90 (0.14)	0,90	0.54–1.38	0.88 (0.12)	0.86	0.62–1.41	0.03
Cu (mg/L)	1.08 (0.18)	1.06	0.46–1.89	1.09 (0.18)	1.07	0.53–1.75	0.60
Cr (mcg/L)	0.46 (0.18)	0.44	0.26–1.23	0.47 (0.25)	0.41	0.01–2.74	0.36
Mo (mcg/L)	2.47 (0.72)	2.41	0.44–6.23	2.48 (0.55)	2.34	0.10–4.53	0.60
Mn (mcg/L)	1.90 (1.17)	1.67	0.12–10.75	1.79 (1.14)	1.11	0.42–8.95	0.13
Se (mcg/L)	106.0 (13.0)	106.30	49.7–138.1	105.2 (11.84)	104.0	74.2–170.0	0.21

**Table 3:** Serum concentrations of heavy metals and bioelements in men with and without type II diabetes mellitus (TIIDM), (AM, arithmetic mean; SD, standard deviation; Med—median; *p*: probability for trend).

Metal	TIIDM (n = 55)			Without TIIDM (n = 258)			*p*
	AM (SD)	Med	Range	AM (SD)	Med	Range	
**Heavy Metals**							
Pb (mcg/L)	72.06 (18.3)	71.60	24.3–112.0	75.05 (22.2)	72.0	26.10–161.5	0.90
Cd (mcg/L)	1.57 (0.30)	1.51	0.59–2.05	1.53 (0.34)	1.57	0.62–3.06	0.46
As (mcg/L)	7.82 (1.35)	7.65	5.10–10.8	7.83 (1.62)	7.92	2.48–15.2	0.98
Hg (mcg/L)	4.47 (0.77)	4.45	0.28–6.60	4.57 (0.88)	4.53	0.15–9.1	0.63
W (mcg/L)	0.61 (0.1)	0.61	0.39–0.74	0.62 (0.1)	0.62	0.28–1.1	0.74
**Bioelements**							
Mg (mg/L)	19.6 (2.25)	19.8	13.8–26.3	21.21 (2.13)	21.15	10.0–37.5	0.0001
Ca (mg/L)	96.9 (5.42)	96.5	83.0–117.0	94.91 (9.79)	95.0	53.5–159.6	0.02
Fe (mg/L)	1.14 (0.41)	1.06	0.53–2.29	1.22 (0.44)	1.45	0.28–3.4	0.11
Zn (mg/L)	0.91 (0.15)	0.89	0.56–1.38	0.88 (0.13)	0.88	0.54–1.41	0.30
Cu (mg/L)	1.06 (0.17)	1.04	0.65–1.41	1.09 (0.18)	1.06	0.46–1.89	0.53
Cr (mcg/L)	0.45 (0.18)	0.40	0.13–1.13	0.47 (0.22)	0.44	0.03–2.74	0.74
Mo (mcg/L)	2.35 (0.75)	2.26	0.11–4.53	2.42 (0.61)	2.39	0.04–6.23	0.87
Mn (mcg/L)	1.75 (1.07)	1.60	0.55–7.50	1.87 (1.15)	1.62	0.12–10.75	0.38
Se (mcg/L)	107.0 (13.5)	106.6	54.1–136.0	105.3 (12.16)	104.5	49.7–170.0	0.21

There were no other statistically significant differences in the levels of the tested elements in men with diabetes compared to those without diabetes. Table 4 presents data on the concentrations of the tested elements in men’s with overweight and obesity compared to those with normal weight. Only Cr and Mn concentrations were higher and lower, respectively, in the men with excess body weight compared to those with normal weight. The concentrations of the other elements were similar in both groups.

**Table 4:** Concentrations of heavy metals and bioelements in men with excess body weight and with normal body weight (AM, arithmetic mean; SD, standard deviation; Med—median; *p*: probability for trend).

Metal	Overweight and Obesity (n = 250)			Normal Body Weight (n = 61)			*p*
	AM (SD)	Med	Range	AM (SD)	Med	Range	
**Heavy Metals**							
Pb (mcg/)	74.80 (21.94)	72.15	24.2–161,5	73.30 (20.0)	71.81	30.77–123.5	0.64
Cd (mcg/)	1.56 (0.33)	1.59	0.59–3.05	1.48 (0.32)	1.53	0.63–2.22	0.56
As (mcg/)	7.79 (1.58)	7.72	3.45–15.2	7.70 (1.69)	7.83	2.47–11.21	0.69
Hg (mcg/)	4.56 (0.87)	4.52	0.19–9.1	4.46 (0.87)	4.49	0.15–6.60	0.82
W (mcg/)	0.61 (0.01)	0.61	0.28–0.83	0.61 (0.01)	0.62	0.28–1.15	0.22
**Bioelements**							
Mg (mg/L)	20.7 (1.81)	20.8	15.7–26.3	21.12 (1.79)	21.15	14.79–26.15	0.41
Ca (mg/L)	96.5 (5.03)	95.3	75.5–109,5	95.15 (8.22)	95.5	53.5–159.6	0.60
Fe (mg/L)	1.17 (0.45)	1.09	0.48–3.40	1.22 (0.43)	1.44	0.28–3.0	0.24
Zn (mg/L)	0.90 (0.12)	0.89	0.56–1.31	0.88 (0.13)	0.87	0.54–1.41	0.14
Cu (mg/L)	1.09 (0.15)	1.07	0.83–1.46	1.08 (0.19)	1.06	0.46–1.89	0.48
Cr (mcg/L)	0.49 (0.19)	0.47	0.15–1.13	0.45 (0.22)	0.41	0.03–2.74	0.03
Mo (mcg/L)	2.45 (0.65)	2.32	0.11–4.53	2.43 (0.64)	2.34	0.04–6.23	0.72
Mn (mcg/L)	2.12 (1.36)	1.79	0.60–8.95	1.74 (1.03)	1.58	0.12–10.75	0.005
Se (mcg/L)	107.4 (13.2)	106.0	54.1–138.1	105.3 (12.1)	104.5	49.7–170.0	0.45

No relationship was found between the concentration of heavy metals and the occurrence of HA (Table 5). An almost similar pattern was observed by comparing bioelements concentrations, with the exception of Mg. It was found that the concentration of Mg was significantly lower in the men with hypertension.

Significant positive correlation coefficients were found for the relations hips W-TCh (r_s_ = 0.14; *p* = 0.017), W-LDL (r_s_ = 0.13; *p* = 0.021), Mg-TCh (r_s_ = 0.25; *p* < 0.001), Mg-LDL (r_s_ = 0.26; *p* < 0.001), Ca-TCh (r_s_ = 0.16; *p* = 0.005), Ca-LDL (r_s_ = 0.11; *p* = 0.049), Ca-insulin (r_s_ = 0.14; *p* = 0.023), Ca-HOMA-IR (r_s_ = 0.13; *p* = 0.041), Zn-TG (r_s_ = 0.22; *p* < 0.001), Zn-insulin (r_s_ = 0.18; *p* = 0.004), Zn-HOMA-IR (r_s_ = 0.19; *p* = 0.002), Cu-BP systolic (r_s_ = 0.11; *p* = 0.05), Mn-BMI (r_s_ = 0.12; *p* = 0.029), Mn-AC (r_s_ = 0.14; *p* = 0.014), Mn-WHR (r_s_= 0.17; *p* = 0.002), Mn-insulin (r_s_ = 0.16; *p* = 0.014), Mn-HOMA-IR (r_s_ = 0.17; *p* = 0.007), Se-TCh (r_s_ = 0.17; *p* = 0.027), Se-LDL (r_s_ = 0.11; *p* = 0.004),Se-TG (r_s_ = 0.19; *p* < 0.001), Se-insulin (r_s_ = 0.19; *p* = 0.002), Se-HOMA-IR (r_s_ = 0.20; *p* = 0.002), Cr-TCh (r_s_ = 0.19; *p* < 0.001), Cr-HDL (r_s_ = 0.14; *p* = 0.012), Cr-LDL (r_s_ = 0.11; *p* = 0.05), Cr-TG (r_s_ = 0.18; *p* = 0.001). Significant negative correlations were found for the relations hip Cd-insulin (r_s_ = −0.14; *p* = 0.02), Hg-WHR (r_s_ = −0.12; *p* = 0.03), W-insulin (r_s_ = −0.29; *p* < 0.001), W-HOMA-IR (r_s_ = −0.25; *p* < 0.001), Mg-BMI (r_s_ = −0.13; *p* = 0.019), Mg-AC (r_s_ = −0.15; *p* = 0.097), Mg-WHR (r_s_ = −0.16; *p* = 0.005), Mg-BP systolic (r_s_ = −0.15; *p* = 0.007), Mo-insulin (r_s_ = −0.12; *p* = 0.05) and Mn-HDL (r_s_ = −0.16; *p* = 0.048) (Table 6). A logistic regression model showed no effect of cigarette smoking on the incidence of MS in the study group (*p* = 0.95).

**Table 5:** Concentrations of heavy metals and bioelements in men with and without hypertension (HA) (AM, arithmetic mean; SD, standard deviation; Med—median; *p*: probability for trend).

Metal	HA (n = 171)			Non-HA (n = 142)			*p*
	AM (SD)	Med	Range	AM (SD)	Med	Range	
**Heavy Metals**							
Pb (mcg/L)	77.07 (21.90)	71.11	26.10–161.50	73.07 (21.30)	74.05	24.30–138.50	0.89
Cd (mcg/L)	1.55 (0.34)	1.57	0.63–3.06	1.54 (0.32)	1.53	0.59–2.24	0.82
As (mcg/L)	7.76 (1.68)	7.80	2.48–15.2	7.92 (1.49)	7.95	3.83–12.5	0.41
Hg (mcg/L)	4.59 (0.89)	4.53	0.15–9.10	4.51 (0.82)	4.45	0.27–7.9	0.25
W (mcg/L)	0.62 (0.01)	0.61	0.15–0.91	0.62 (0.01)	0.63	0.39–0.83	0.11
**Bioelements**							
Mg (mg/L)	20.58 (2.59)	20.5	10.0–37.5	21.36 (1.61)	21.3	16.6–26.3	0.00012
Ca (mg/L)	95.39 (8.93)	95.0	53.5–159.6	95.10 (5.19)	95.5	77.0–109.5	0.72
Fe (mg/L)	1.21 (0.46)	1.07	0.29–3.40	1.21 (0.40)	1.25	0.28–2.38	0.79
Zn (mg/L)	0.88 (0.14)	0.88	0.54–1.41	0.89 (0.12)	0.88	0.64–1.29	0.20
Cu (mg/L)	1.08 (0.19)	1.06	0.46–1.89	1.07 (0.16)	1.06	0.53–1.46	0.77
Cr (mcg/L)	0.44 (0.18)	0.42	0.26–3.17	0.49 (0.25)	0.44	0.02–2.74	0.09
Mo (mcg/L)	2.39 (0.64)	2.32	0.44–4.39	2.50 (0.63)	2.43	1.35–6.23	0.16
Mn (mcg/L)	1.86 (1.12)	1.62	0.12–10.75	1.83 (1.06)	1.63	0.42–8.95	0.85
Se (mcg/L)	105.6 (13.9)	104.5	49.7–170.0	105.7 (10.2)	105.0	74.2–136.0	0.86

## 4. Discussion

### 4.1. Heavy Metals

In this study we found no statistically significant differences in the concentrations of heavy metals (Pb, Cd, Hg, As and W) between the men with MS (as well as those with the components of the MS, such as hypertension, obesity and diabetes) and healthy participants. This may be due to cut between having MS and not depends on levels of a number of markers and is not a uniform cut off, so risk prediction is not very accurate [38]. However, the correlation coefficients of all participants together was most negative (r = −0.29 and −0.25, *p* < 0.001) for W–Insulin and W-Homa-IR of all analyses made in this study, and there was also a week correlation with Cd and insulin. Whether this was a toxic effect of W and Cd on pancreatic cells or some other is not clear. Decreased pancreatic insulin reserve and action could precipitate hypo-insulinaemic diabetes, and late stage TIIDM. The positive correlation which we observed between W and TCh and LDL levels further suggests that a higher W may be atherogenic, as confirmed by epidemiological studies. This can be explained by the fact that in the etiology of atherosclerosis an important role is played by oxidative stress and inflammation, and W is pro-inflammatory and involved in the production of free radicals (as the component of oxidoreducates). Some results report that environmental exposure to W intoxication may contribute to the development of cardiovascular and cerebrovascular diseases [39,40]. West Pomerania is an agricultural and industrial region. The main branch of the economy is agriculture and the food industry. Other important industries are: timber, metallurgy, chemicals, shipbuilding, and the production of electricity. Environmental conditions in the region have been improving for many years. Emissions of pollutants into the air from point sources (industry, energy) have tended to decline. This is related to ecological investment, mainly in the energy sector (report on the state of the city of Szczecin, 2012). At the same time a large part of the region is legally protected under the Wildlife Protection Act (Dz. U. 2004, No. 92, item. 880), and some areas are part of the European Ecological Network Natura 2000—Special Protection Areas—Wkrzańska refuge, Świdwie Lake, Goleniów Forest and Skoszew Grasslands (Dz. U. 2008, No. 198, item. 1226); those areas at least partially overlap with the area of research [41]. Thus these older men may be showing evidence of past accumulated exposure to heavy metals from the above industry with inadequate pollution management or perhaps there is still industrial residue in the areas under horticulture.

### 4.2. Bioelements

In this study, Mg levels in participants with MS were significantly lower than in those without MS, which is consistent with the research of Guerrero-Romero *et al.* [42,43]. We also found that our participants from north-western Poland with diabetes and hypertension had significantly lower Mg concentration compared to those without these conditions. The results of this study regarding participants with diabetes are consistent with the results of other authors [42,44]. Sun *et al.* [31] indicate a reduced Mg concentration in people with MS and type 2 TIIDM, and a statistically significant negative correlation between Mg concentration and the coefficient of insulin resistance [31]. These authors also report significantly lower Mg concentration in people with overweight and obesity. In our study this relationship was not observed. Choi *et al.* [45] demonstrated that participants with MS had a lower intake of Mg than the control group. Our results show that the greater the abdominal circumference, BMI, WHR and BP, the lower Mg concentration in the serum, which may result from a high-fat and high-carbohydrate diet.. Likewise, Sun *et al.* [31] indicate a statistically significant negative correlation between Mg concentration and both BMI and WHR in the participants. On the other hand, our study found positive correlations between the concentrations of Mg to TCh or LDL, which is consistent with studies of Nasri *et al.* [46]. The dependencies and correlations presented between Mg concentration and the analyzed parameters may suggest that Mg does not protect against disorders of lipid metabolism but may have a beneficial effect on blood pressure, carbohydrate metabolism, and reduce the risk of abdominal obesity. In contrast, Sun *et al.* [31] present no relationship between Mg concentration and lipid parameters in a study conducted with both men and women. Mak *et al.* [47] report that Mg supplementation improves the lipid profile and thus helps to reduce the risk of cardiovascular disease.

In this study we found that Ca may be associated with impaired lipid and carbohydrate metabolism, as evidenced by the positive correlations between the concentrations of this element and the concentrations of TCh, LDL, insulin and HOMA-IR. Furthermore, statistically significantly higher Ca concentration were found in the participants with diabetes. The results by Sun *et al.* [31] on lipid metabolism are similar to those presented here, but the authors found no correlation between Mg concentration and the parameters of carbohydrate metabolism. Likewise, Park *et al.* [48] report a statistically significant correlation between Ca concentration and the occurrence of MS in Korean men aged from 30 to 60 years.

The men with MS from the study group indicated a significantly higher Zn concentration than those without MS. None of the study participants had symptoms of Zn deficiency. Due to the positive correlation between Zn and insulin concentrations as well as HOMA-IR in the examined participants without diabetes, lower Zn levels may relate to disturbance of carbohydrate metabolism. The role of Zn in carbohydrate metabolism is complex. Zinc plays an important role in synthesis, storage and release of insulin [49]. This element affects the functioning of the beta cells in the islets of Langerhans. Animal research shows that Zn administered in small doses protects against type 2 diabetes but a high concentration of the element is toxic to the beta cells in the islets of Langerhans [50]. In this study we also observed a positive correlation between Zn and TG concentrations., similar to Seo *et al.* [51] and Ghasemi *et al.* [52]. Research by Seo *et al.* [51] suggests that high serum zinc levels might be associated with metabolic risk factors (TG and waist circumference) in men, but not in women. Obeid *et al.* [30] report no statistically significant correlation between the concentration of Zn and MS with its individual components in adults from Lebanon aged 18-65 years. Ahn *et al.* [53] also find no connection between the occurrence of MS and Zn concentration and indicate a negative correlation between the concentration of this element and HOMA-IR. 

With respect to Cu we found that the men in our study indicated a positive correlation on the verge of statistical significance with and systolic and diastolic BP. We observed no correlation between the concentration of this element and other parameters of MS. The findings of other authors suggest that Cu concentration in patients with HA is significantly higher [54]. Afridi *et al.* [55] show that deficiency of Cu is synergistically associated with other risk factors for high blood pressure. Carpenter *et al.* [56] on the basis of results of other authors on the role of Cu in HA, argue it is difficult to draw definite conclusions due to the lack of homogeneity of the study groups. 

Drawing on the findings of other authors, Onapkoya *et al.* [57] report that Cr supplementation causes weight loss, but the effect is small and of uncertain clinical significance. Indeed, our study revealed that Cr concentration in men with obesity and overweight was significantly higher and a positive correlation between Cr concentration and BMI was of borderline statistical significance (*p* = 0.057). Similarly, in women from southern Poland a statistically significant positive correlation existed between Cr concentration and BMI [58]. In contrast, Yerlikaya *et al.* [59] report no significant differences in Cr concentration in obese women compared to the control group. In the study presented here, we found an association between Cr concentration and lipid metabolism parameters, but there was no such relationship for carbohydrate metabolism. Likewise, Sun *et al.* [31] indicate a relationship between the concentrations of Cr and lipid components with the exception of HDL. These researchers also show statistically significant positive correlations between the concentrations of Cr to insulin and HOMA-IR. Kim *et al.* [60] indicate that in men with visceral obesity Cr level in hair is positively correlated with insulin resistance, and Chung *et al.* [61] report lower Cr level in the hair of men with MS.

**Table 6:** Spearman correlation coefficients between element levels and metabolic features (BMI, Body Mass index; AB, abdominal circumference; WHR, waist-to-hip ratio; BP, blood pressure; TCh, total cholesterol; HDL, high-density lipoprotein; LDL, low-density lipoprotein; TG, triglyceride; HOMA-IR, Homeostasis Model Assessment—Insulin resistance).

Metabolic Features	Pb	Cd	Hg	As	W	Mg	Ca	Fe	Zn	Cu	Mo	Mn	Se	Cr
BMI	0.02	0.06	0.57	−0.03	0.01	−0.13 *	0.01	−0.09	0.08	0.02	−0.019	0.12 *	0.03	0.12
AB	0.04	0.06	0.01	−0.03	−0.04	−0.15 *	0.03	−0.08	0.06	0.05	0.04	0.14 *	0.07	0.11
WHR	−0.07	−0.07	−0.12 *	−0.08	−0.14 *	−0.16 *	0.008	−0.05	0.003	0.07	0.004	0.17 *	0.04	0.04
BP systolic	0.06	0.04	−0.02	0.07	−0.06	−0.15 *	−0.0007	0.04	0.001	0.11 *	0.06	−0.019	0.07	−0.004
BP diastolic	0.04	−0.02	−0.02	0.04	−0.11	−0.0004	0.04	0.04	0.08	0.10	0.1	0.03	0.11	0.02
TCh	−0.08	−0.001	−0.04	−0.04	0.14 *	0.25 *	0.16 *	−0.02	0.09	0.04	0.03	0.06	0.17 *	0.19 *
HDL	−0.07	−0.03	−0.09	0.04	0.9	0.009	0.02	0.09	−0.07	−0.05	0.05	−0.16 *	−0.05	0.14 *
LDL	−0.03	0.04	−0.02	−0.03	0.13 *	0.26 *	0.11 *	−0.04	0.03	0.04	−0.007	0.09	0.11 *	0.11 *
TG	−0.07	−0.07	0.04	−0.04	−0.05	−0.06	0.098	−0.05	0.22 *	0.05	0.03	0.02	0.19 *	0.18 *
Insulin	−0.06	−0.14 *	0.01	−0.06	−0.29 *	−0.019	0.14 *	−0.07	0.18 *	0.06	−0.12 *	0.16 *	0.19 *	−0.09
HOMA-IR	−0.07	−0.11	0.004	−0.09	−0.25 *	−0.056	0.13 *	−0.02	0.19 *	0.04	−0.96	0.17 *	0.20 *	−0.08
FPG	−0.05	0.02	0.04	−0.05	0.06	−0.11	−0.4	0.09	0.04	−0.08	0.05	0.05	−0.005	−0.05

* *p* ≤ 0.05.

We found a relationship between Se concentration and lipid metabolism (with the exception of HDL) and carbohydrate parameters (insulin and HOMA-IR), which suggests that higher concentrations of this element may contribute to metabolic disorders. Similar results were obtained by Kim *et al.* [60] and Sun *et al.* [31]. On the other hand, Aranud *et al.* [62] report that in women with MS but without TIIDM Se concentration is higher, while in men no such correlation is observed. Furthermore, Obeid *et al.* [30] indicate positive correlations between Se concentration and the values of all measured parameters characteristic of MS. The results of this study and other authors suggest that Se may contribute to the development of MS.

In the present study, we found a statistically significant higher Mn concentration in the obese men and observed a positive correlation between Mn concentration and the anthropometry and parameters of carbohydrate metabolism (insulin, HOMA-IR), and a negative correlation between the concentrations of Mn and HDL. In contrast, Al-Sabaawy [63] reported that Mn concentration in both the men with dyslipidemia and the control group were similar. Likewise, no statistically significant correlation between the concentration of Mn and lipid parameters was observed by Sun *et al.* [31]. Those researchers also pointed out a statistically significant negative correlation between Mn concentration and carbohydrate metabolism. Forte *et al.* [64] showed that Mn concentration in people with diabetes mellitus type 1 and 2 was lower than in healthy participants. Koh *et al.* [65] suggest that Mn may play a protective role in diabetes. On the other hand, no such relationship was present in seniors from the Czech Republic [66].

In available literature, there is little data on the relationship between Mo and metabolic disorders. Flores *et al.* [67] show that antagonistic interactions between Mo and Cu may increase the progression of diabetic complications. In the present study, as well as in the research of Sun *et al.* [31], there was a positive correlation between the concentrations of Mo and insulin. Moreover, Sun *et al.* [31] indicate a statistically significant positive correlation between Mo concentration and HOMA-IR. The results of our and other authors’ studies may indicate a role of Mo in the formation of carbohydrate metabolism disorders.

The results of this study and those of other authors on the relationship between bioelements and MS with its components and other metabolic parameters are inconclusive. Therefore, mineral supplementation cannot be recommended as a means of prevention of metabolic disorders. Also Czernichow *et al.* [68] question the benefits of supplementation with minerals, including antioxidants, despite the lack of side effects. For that reason, comprehensive total diet analyses, including plant and animal based foods, should be run on the population Additional and methodologically comprehensive population-based (modeling) studies should be carried out to further determine the significance of macro- and microelements for human health.

## 5. Conclusions

In the studied men aged 50–75 years who had not been knowingly exposed to heavy metals in their work or living environment, the data showed that higher W concentrations may be a contributing factor to dyslipidaemia. Among the macroelements, Mg seemed to have a protective role in the occurrence of metabolic disorders, especially through beneficial effects on carbohydrate metabolism, blood pressure and anthropometric parameters. Higher concentrations of Ca in diabetics and positive correlations with HOMA-IR and insulin levels may indicate a detrimental effect of this mineral on carbohydrate metabolism. Microelements such as Mn, Cr and Se may contribute to the incidence of metabolic disorders. However, it was not possible to unambiguously assess their relevance to the formation, occurrence and severity of metabolic disorders in men. 

## Abbreviations

MS: Metabolic syndrome
TIIDM: Type II diabetes mellitus
BMI: Body mass index
AC: Abdominal circumference
WHR: Waist-to-hip ratio
TCh: Total cholesterol
LDL: Low-density lipoprotein
HDL: High-density lipoprotein
TG: Triglyceride
FPG: Fasting plasma glucose
HOMA-IR: Homeostasis Model Assessment – Insulin resistance
IR: Insulin resistance
BP: Blood pressure
HA: Hypertension
Pb: Lead
Hg: Mercury
As: Arsenic
Cd: Cadmium
W: Tungsten
Mg: Magnesium
Fe: Iron
Ca: Calcium
Zn: Zinc
Cu: Copper
Se: Selenium
Mn: Manganese
Cr: Chromium
Mo: Molybdenum
